# Efficient Switches in Biology and Computer Science

**DOI:** 10.1371/journal.pcbi.1005100

**Published:** 2017-01-05

**Authors:** Luca Cardelli, Rosa D. Hernansaiz-Ballesteros, Neil Dalchau, Attila Csikász-Nagy

**Affiliations:** 1 Microsoft Research, Cambridge, United Kingdom; 2 Department of Computer Science, University of Oxford, Oxford, United Kingdom; 3 Randall Division of Cell and Molecular Biophysics, King's College London, London, United Kingdom; 4 Pázmány Péter Catholic University, Faculty of Information Technology and Bionics, Budapest, Hungary; ETH Zurich, SWITZERLAND

## Introduction

Biological systems are adapted to respond quickly to changes in their environment. Signal processing often leads to all-or-none switch-like activation of downstream pathways. Such biological switches are based on molecular interactions that form positive feedback loops. Proper signal processing and switching have to be made by the noisy interactions of fluctuating molecular components; still, switching has to happen quickly once a threshold in the input signal is reached. Several computing algorithms have been designed to perform similar all-or-none decisions with high efficiency. We discuss here how the structure and dynamical features of a computational algorithm resemble the behaviour of a large class of biological switches and what makes them work efficiently. Furthermore, we highlight what biologists can learn by looking at specific features of computational algorithms.

## Computer Science Is Influencing Our Thinking about Biology

In the 20th century, biological systems were mainly studied from a reductionist perspective through experimental and observational approaches [1,2]. In this period, biology was focused on genetics and later on molecular biology, investigating individual entities, such as genes or proteins, one at a time. In the 19th century, an integrative perspective emerged in physiology through the works of Ivan Pavlov and Claude Bernard [3,4]. However, in the context of biology, systems approaches were first mentioned only in the mid-20th century [5–7], and spread in the first years of the 21st century. This spread was thanks to the impact of mathematics and physics on biological thinking [8,9] and to technological advances that now enable biologists to do genome- and proteome-wide measurements [10]. Systems biology eventually helped us in making predictions about structural and dynamical features of biological systems and in driving experimental studies. Since then, systems biology has become a distinct research field [11,12] and a “new prism through which biology can be understood” [1]: biology cannot be understood as a sum of individual biological entities, but by investigating the complex behaviours arising from their interactions [8].

**Fig 1:**
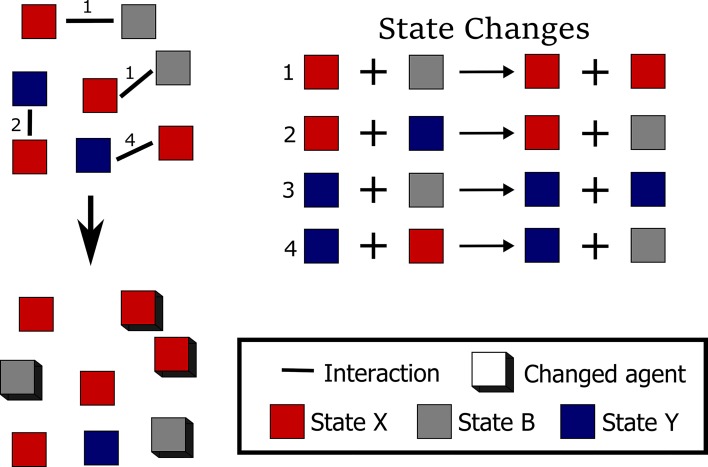
Population protocols. A communication protocol is, in general, a set of rules about how a set of agents can interact. In population protocols, in particular, these rules very closely mirror assumptions about chemical solutions. Namely, agents interact only in pairs (like in a molecular collision), the next pair to interact is chosen randomly (like in a well-mixed solution), each agent can have only a finite number of states (like phosphorylation states), and each interaction can result in a change of state in either or both agents. It is therefore easy to draw a parallel between agent states and chemical species (interacting molecules) and between binary interactions and bimolecular reactions (unimolecular reactions can be handled as a special case). In that way, population algorithms can be translated into chemical reaction networks and vice versa [23]. Population protocols are also used at a different abstraction level, modelling interactions between species of a biological population [24]. The set of four state changes in this figure implements the Approximate Majority population protocol. Each square is an agent in one of its three possible states (colours). Pairs of agents have the potential to interact according to the left-hand-side patterns of the state changes and produce new states according to the corresponding right-hand-side patterns. The interactions that actually happen are determined randomly, but starting from the configuration on the top left they may result in four steps in the new configuration on the bottom left, where further interactions become possible.

This field has continued to grow and expand, taking influence from other disciplines. Computer science has contributed to the establishment of new tools to process the massive increase of biological data [13] and to formal approaches for the accurate representation of biological knowledge in a computable form [14,15]. Special interest has grown around the application of theoretical computer science and algorithms to biology [15–18]. Theoretical computer science contributes by analysing the correctness of algorithms (how they exhibit global system properties) and by analysing their intrinsic efficiency (distinguishing classes of algorithms up to multiplicative constants, hence independently of precise parameters [19]). Specific algorithms can be used to explain biological mechanisms, as many of them have counterparts in nature, including those for network routing, distributed search, and consensus establishment [18]. The parallels happen particularly in situations in which there are many independent computational entities that, similarly to collections of molecules or organisms, interact and cooperate through chance encounters. Particular examples are population protocols (Fig 1) [20]. As with the parallels between ecological and molecular networks [21,22], here we claim that population protocols can help us to understand a specific class of basic biological functions.

The algorithmic viewpoint, exemplified here by population protocols, will become increasingly important in the analysis of biological systems. As we increase our detailed understanding of biological networks, understanding their complex information flows will gradually take precedence over biochemical details that may be more accidental. We need to understand not only what molecular states exist but also what they represent with respect to specific tasks that a cell needs to perform. A given phosphorylation state inside a cell may represent some environmental condition detected outside the cell: the logical interactions between these representations are what we need to track, beyond the underlying molecular interactions.

But how do we precisely relate a (biochemical) network, the components of which are molecules and reactions, to a (computational) algorithm, the components of which are symbolic properties and events? We can in fact reduce this as the task of comparing the functionality of different networks: these can be all biochemical networks or a mixture of biochemical and algorithmic networks. To this end, we will discuss the notion of network emulation, by which a network can, in a specific sense, impersonate the functionality of another network that is easier to analyse. Comparing networks and algorithms in such a way is actually a frequent activity in computer science but is mostly used for discrete systems such as software programs and communication protocols [25]. Here, we focus on continuous versions of those notions that are more appropriate for the analysis of dynamical system properties and hence for biochemical networks. We expect that the sophistication and usefulness of techniques for comparing network functionality will grow in the future.

## The Cell Cycle Switch as an Algorithm

Computer science and biology can influence each other deeply because there are many parallels between carefully developed algorithms and highly evolved biological systems [18]. An example of such a parallel is the similarity in the behaviour of the Approximate Majority (AM) algorithm with the dynamical features of the cell cycle G2/M transition regulatory network [26]. AM is a fast population protocol [27] describing how to drive a population of agents that are initially in either of two states (X or Y) into a final population in which all agents are in the same state (Fig 1). That is, the aim is to achieve consensus through the whole population, and the algorithm guarantees that the initial majority of X or Y will almost certainly win out in the end. The algorithm is “approximate” because it is inherently stochastic and there is always some (small) probability that the initial majority will lose, particularly if the two initial populations have similar sizes. On the other hand, stochasticity guarantees that a consensus will always be reached eventually.

**Fig 2:**
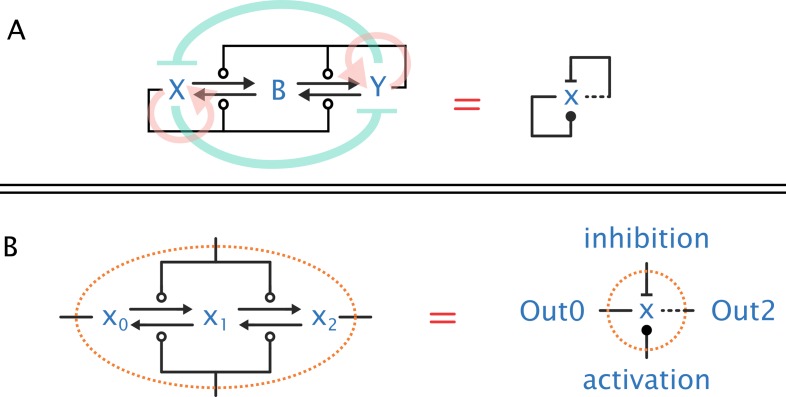
AM and network notation. (A) We recast the AM algorithm of Fig 1 as a wiring diagram. Left: the four reaction arrows correspond to the four state changes in Fig 1 (the hollow circle stands for a catalytic reaction). The system presents two active states (X and Y) that are antagonist: X activates itself and inhibits Y, and Y activates itself and inhibits X. Background arrows indicate the generated feedbacks loops: red arrows represent the pure positive feedback loops and green ones represent the antagonistic double-negative, thus also positive, feedback loop. Right: a condensed representation of the same network according to the abbreviation explained in (B). The condensed graph shows that x activates itself (solid line indicates X’s actions) and inhibits itself (dashed line indicates Y’s actions). (B) Notation for condensed influence networks. A node X (right diagram) represents three species (x_0_, x_1_, x_2_) and four reactions (left diagram). A node X influences other nodes when it is in either of its extremal states: 0 state (Out0—solid outgoing edge) or 2 state (Out2—dashed outgoing edge). A node can be activated (ball-end edge) or inhibited (bar-end edge) by other nodes, the reactions of which drive the node between its various states. Note how the collapsed notation in (B) collapses the network (A, left) into the network (A, right). In the sequel, we mostly draw collapsed networks, which can be systematically expanded.

There can be many ways to achieve consensus, and some may be faster than others. The AM algorithm trades off accuracy in favour of a fast runtime [27], in contrast to other algorithms that are exact but slower or use more resources [28]. The AM algorithm employs a third undecided agent state (B) in addition to the decided agent states X and Y. We can explain the algorithm directly in terms of chemical reactions: (1) if two agents of opposite decided states meet, one of them randomly becomes undecided (X + Y → X + B, X + Y → B + Y), (2) if an undecided agent meets a decided agent, it takes on the state of the decided agent (X + B → X + X, B + Y → Y + Y), and (3) otherwise nothing changes. Note that the two decided states are antagonistic, as they move opposing agents away from the other decided state. At the same time, both of these states are autocatalytic; thus, in AM there are three positive feedback loops: two pure positive and one antagonistic (Fig 2) [29].

In a biological context, feedback loops are responsible for most complex dynamic behaviours [30–32]. If we consider two molecules, X and Y, that can influence each other’s activity or level, then depending on the effects of interactions (negatives or positives), they can form two types of feedback loops. Negative feedback loops contain an odd number of negative interactions (i.e., X positivity affects Y, and Y negatively affects X), which can help to keep the system in homeostasis. Longer negative feedback loops, which produce delayed self-inhibition, can induce oscillations [33,34]. On the other hand, positive feedback loops contain only regulatory interactions with positive effects or an even number of negative interactions (i.e., in the two molecules case X and Y, both negatively affect each other, leading to an antagonism between X and Y). These architectures can create multistability: at a given level of external input, the system can exist in multiple states, and only its history determines in which states it sits. When the external input changes, the system can go through a bifurcation; then, one or more of these steady states disappear, so if the system was resting in such a state, it will transition to a different one. This is the basis of biological switches: they can convert a graded input change into a digital on/off switch when a critical threshold in the input signal is reached [35,36]. Strikingly, once the switch is turned on/off, it should rest in this state irrespective of noise in the signal.

**Fig 3:**
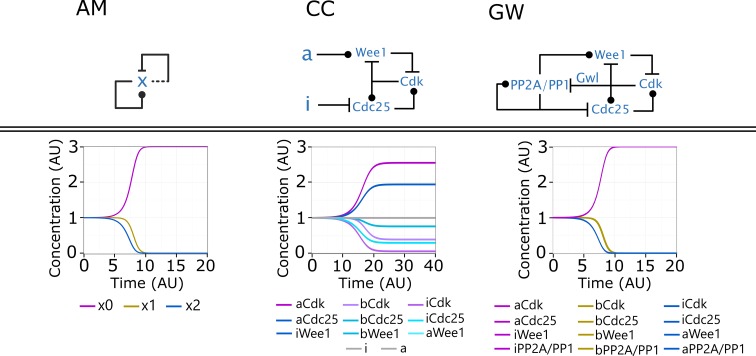
Similarity in switching dynamics between approximate majority (AM) and two cell cycle model systems. Top: AM, represented as an influence network in the notation of Fig 2. CC: The classical cell cycle module of the G2/M transition regulation, represented as a molecular level wiring diagram in the notation of Fig 2, with a and i serving as proxies for a constant phosphatase counteracting the effects of Cdk. GW: The cell cycle model extended with the regulation of phosphatases (PP1 and PP2A) through the Greatwall kinase pathway [45,46], represented as molecular wiring diagram. Bottom: Examples of the deterministic behaviour of each network initiated from an undecided state in which all species are present in similar amounts. The AM and the GW systems show equal dynamics. Only the various forms of Cdk of the CC system resemble the behaviour of AM and GW, but because of the external influence of a and i, the other species do not overlap with these while they properly align with them in GW.

Perhaps the best characterized biological switch is the one that controls the critical cell cycle transition from interphase (G2 phase) to mitosis (M phase). This transition is driven by an abrupt switch in the activity of the mitotic cyclin-dependent kinase (Cdk) complex [37]. At the G2/M transition, Cdk is activating its activator Cdc25 while it is inhibiting its inhibitor Wee1 (Fig 3) [38]. These two positive feedback loops (one pure positive, one antagonistic) are driven by phosphorylation and dephosphorylation events on multiple sites on each of these proteins [39,40]. The nonlinearity caused by the multisite regulation and the presence of positive feedback loops cause this system to behave like a bistable switch [41,42].

Comparing AM (Fig 2) with the classical G2/M regulatory module (CC on Fig 3), we can see that they do not look much alike. They have a different number of species and their regulation seems different, although both AM and CC contain several positive feedback loops combined with multistep transitions between active and inactive states. Despite the differences, we have recently shown that the steady state solutions at various inputs and switching dynamics of the AM and CC model follow similar patterns [28]. This similarity was observed even when both models were simulated with the simplest possible parameter set (all reaction rates equal to one), but this can be extended to other parameter sets [43]; moreover, there are analytical explanations for the similarity [44].

Although the behaviour of Cdk in the CC model preserves most of the dynamical features of X in AM, the upper steady state in the CC model does not reach maxima, and the transition between the two states is slower (Fig 3). This difference is caused by the external signals (a and i), which continuously act against Cdk, serving as thresholds against Cdk and forcing Cdc25 and Wee1 into their other steady states [26]. But this difference is essentially an artefact of the early models of G2/M: in a fuller model, these reactions are controlled by the phosphatases PP2A and PP1, which in turn are inhibited by Cdk through the Greatwall kinase (Fig 3) [45,46]. This extended “GW” network has more positive feedback loops than CC, because PP2A/PP1 and Cdk are mutually antagonistic, and PP2A/PP1 is autocatalytic (Fig 3). In the absence of a continuous pressure on Cdk, the resulting system shows a full transition to the upper state and now exactly matches AM deterministic kinetics (see S1 Text) [44]. Also note that with this basal parameter setting of all kinetic rates equal to one, even all forms of Cdc25, Wee1, and PP2A behave similarly to the various forms of X in AM (see more on this later). Confirming this computational finding [26], it was recently experimentally shown that Greatwall is indeed essential for a complete switch into mitosis in frog and starfish eggs [47]. We have also shown that the speed of the transitions in the GW model is as fast as those of the AM model [26]. Because of the known algorithmic properties of AM [27], we can conclude that the extended (GW) model can drive a fast and complete cell cycle switch at the G2/M phase.

**Fig 4:**
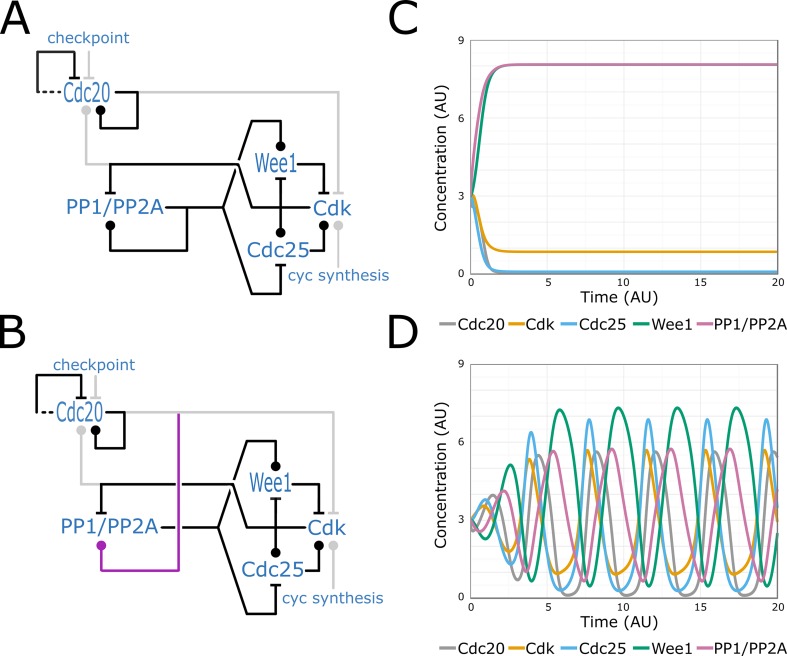
Cell cycle oscillations controlled by the GW switch. The extended GW model of Fig 3B is embedded into the negative feedback loop in which Cdk activates Cdc20 and by this induces its own removal. (A) Model with autocatalytic activation of PP1/PP2A. (B) Model with PP1/PP2A activated by Cdc20. (C, D) Simulations of the models on A and B, respectively. On the left panels, the GW model is drawn in black, the negative feedback loop components in light grey (following [26]), and the new proposed interaction in purple.

We now consider the cell cycle switch in the wider context of cell division. Every time a cell undergoes a new cycle of division, the G2/M transition takes place. In biological systems, we can observe sustained and robust oscillatory behaviours when negative feedbacks loops are combined with positive feedbacks [48,49]. This idea was used to connect the minimal switching model AM, or the basic CC model, to a negative feedback loop, and, as expected, these systems showed robust oscillations [26]. If we similarly connect the GW model to a negative feedback loop, representing the activation of the complex of Cdc20 and the anaphase promoting complex (APC), which eventually degrades cyclin [50], then we do not observe oscillations (Fig 4A and 4C). The reason for this is that the direct positive feedback loop on PP2A/PP1 can maintain the high phosphatase and low CDK activity even after Cdc20 level dropped. This differs from AM, in which a lower number of shorter positive feedback loops are coupled in a way that ensures all molecules switch between their alternative states at the same time. We resolve this lack of oscillations if the phosphatases receive an initial trigger from Cdc20, but they cannot maintain their high activity when Cdc20 levels drop. Thus, the introduction of such a link from Cdc20/APC to phosphatase activation leads to a system with robust limit-cycle oscillations (Fig 4B and 4D). Although there is no evidence for such a direct link between Cdc20 and PP2A in higher eukaryotes, there are data showing Cdc20 induces indirect activation of PP1 [51].

## A Class of Efficient Biological Switches

The main requirements for an optimal switch are that a system should find a steady state quickly (it should be efficient), even from a random initial undecided state (it should be reliable in finding steady states), and it should return to this steady state after small perturbations in the variables of the system (it should be robust). Theoretically, AM has been shown to be an efficient switching algorithm, in the sense of converging quickly to one of its steady states from any initial state (and thus being reliable), and has a strong robustness against noise as well [27]. By relating the kinetics of biological switches to the behaviour of AM, we can use known theoretical results to immediately conclude that biological switches are efficient too [26], in addition to being reliable and robust (which was largely known [52,53]).

Despite the extensive literature on biological switches [54–56], it still remains unclear what actually makes biological switches efficient. In fact, it is not even well established whether efficiency is useful in the context of a biological switch: the speed of biological switches is far less investigated than their steady state dynamics, and it is not clear how far biological systems are optimized for fast switching [18]. It has already been proposed that interlinked positive feedback loops on different timescales can speed up switches [55]. Obviously, slow switching will at some point be deleterious, but another consideration is that biological switches are often assembled dynamically and disassembled shortly after [57]. Hence, the initial state of a freshly assembled protein complex controlling a biological switch can be random or undetermined and needs to settle quickly into the “default” state from any initial conditions. During the progress of the cell cycle, various switches are setting appropriate initial conditions for the following switch, ensuring that each cell cycle transition is controlled and kept in proper order [58], with the actual Cdk activity level serving as a cue for the initial state of each switch.

**Fig 5:**
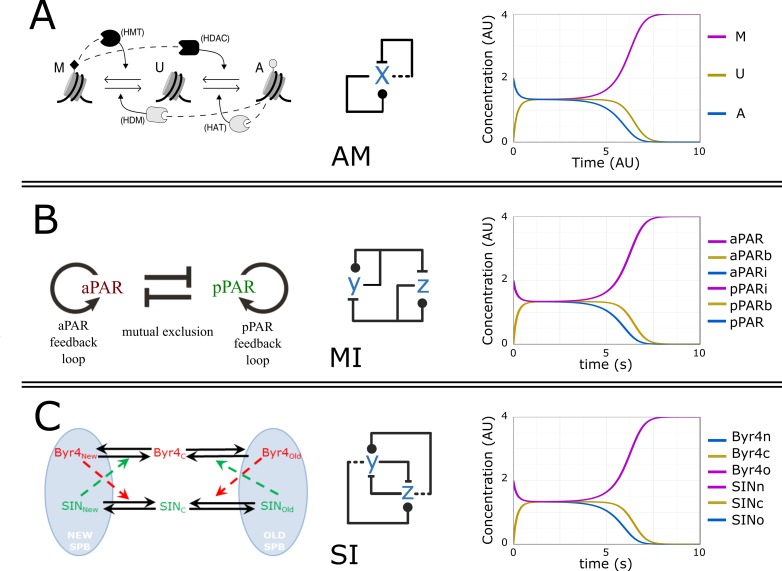
Biological networks with switching dynamics. (A) The epigenetic switch model proposed by Dodd et al. (2007) [61]. (B) The polarity regulatory model of Motegi et al. (2013) [63]. (C) The septation initiation network asymmetry establishment model of Bajpai et al. (2013) [64]. The middle panels show the respective models with the condensed network notation, in which each node (molecule) represents three forms: inactive, non-decided, and active (Fig 2); the right panels show the behaviour of the models when initiated from equal initial conditions and simulated with equal parameter values (all rates = 1). On panels B and C, only three traces are visible, as they totally overlap with the other three traces.

The G2/M transition of the cell cycle is a classic example of an important biological switch, but several other biological switches, including the control of other cell cycle transitions [59], work with similarly structured regulatory networks, containing multiple positive feedback loops [60]). Strikingly, the epigenetic memory switch [61] shows an exact structural equivalence with the AM model. Each nucleosome can exist in one of three states: methylated (M), unmodified (U) or acetylated (A). Histone-modifying enzymes (HMT, HDM, HAT, and HDAC) are recruited by modified nucleosomes to interconvert nearby unmodified ones. A whole population of histones thus switches between methylation/acetylation states, generating a switch-like behaviour exactly matching that of the AM network (Fig 5A). Other highly investigated biological switches with two positive feedback loops include the lambda phage lytic-lysogenic switch [62], polarity establishment [63], and symmetry breaking [64]. Among these, the asymmetric activation of the septation initiation network in fission yeast shows a network structure (SI) that contains a double positive and a double negative feedback loop, leading to the exact same dynamics as AM (Fig 5C). The polarity establishment by the PAR system contains two positive feedback loops and one double negative feedback loop, which resembles a minimal model (MI) of two antagonistic but at the same time autocatalytic molecules (Fig 5B). All these networks function through multiple positive feedback loops and are driven by posttranslational modifications on multiple sites, resembling the basic features of the efficient AM algorithmic switch. Furthermore, they all show very similar dynamical behaviour.

Models of biological switches can show equally fast switching independent of their exact wiring, at least for some choice of parameters (in these examples, all rates = 1; Fig 5). Thus, the separation of the timescales of the two positive feedback loops [65] might be less important than the presence of multiple loops. Interestingly, the simulations of the models of Fig 5 are not only similarly fast but they show the exact same dynamics, suggesting that these biological switches form a special class with equal dynamical features and switching efficiency.

## Different Network Structures, Similar Dynamic Solutions

In the context of cell cycle regulation, it was pointed out that network topology and dynamical behaviour are better conserved than sequences of key molecules [66]. But, as we have seen above (Figs 3 and 5), sometimes even the exact network structure can be different, while the dynamical behaviour can be preserved.

**Fig 6:**
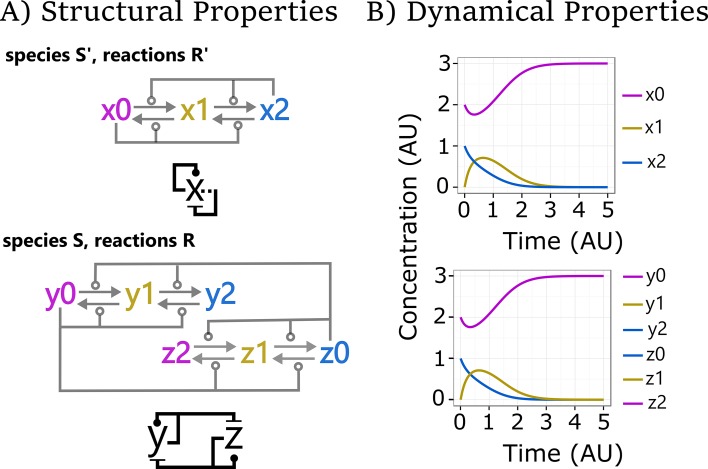
Network emulation. Condensed and extended wiring diagrams of AM (above) and MI (below) and their deterministic behaviour in time-course diagrams. A morphism *m*:(S,R)→(S’,R’) between two reaction networks (S,R) and (S’,R’) is a mapping of species S (e.g., y_0_, y_1_, y_2,_ z_0_, z_1_, z_2_ of MI) to species S’ (e.g., x_0_, x_1_, x_2_ of AM, by corresponding colours) and of reactions R to reactions R’. Structural properties: A morphism that preserves the reactants and products of each reaction under the mapping is called a homomorphism. One that preserves stoichiometry under the mapping (by appropriately summing multiplicities and rates) is called a stoichiomorphism. These properties can be calculated directly on the network representation. Dynamical properties: A morphism *m* is an emulation if it preserves all trajectories of species concentrations over time under the mapping (e.g., the trajectories on the right are preserved). That is, *m* is an emulation if for any choice of initial conditions I’ for S’ there exist initial conditions I for S such that the trajectory of each species s in S overlaps exactly the trajectory of *m*(s) in S’. Theorem [44]: A morphism that is a homomorphism and a stoichiomorphism is also an emulation.

**Fig 7:**
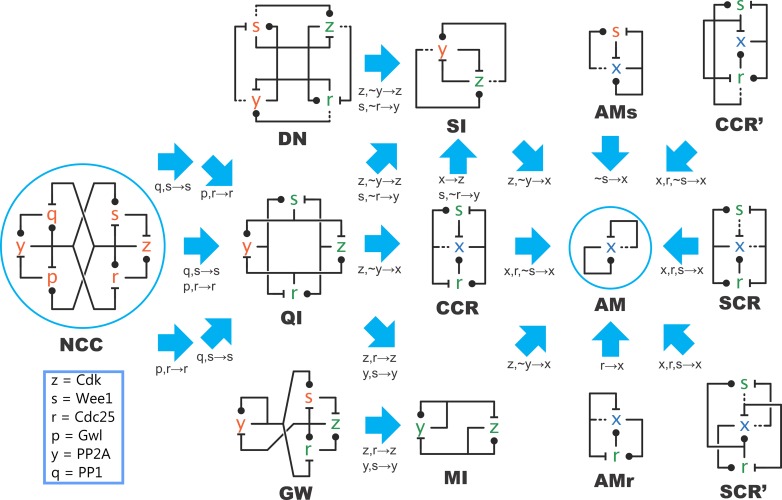
Network morphisms. Blue arrows are both homomorphisms and stoichiomorphisms, implying kinetic emulation. The species mapping is indicated under each arrow; the reaction mapping is the associated homomorphic projection that simply respects the species mapping. A mapping like ~y→x means that the species y_0_,y_1_,y_2_ are mapped respectively to x_2_,x_1_,x_0_ (based on the notation of Fig 2). Molecular names are indicated for the NCC network, which can be mapped back to some of the more basic ones (like GW and CCR), but not to all. Figure is adapted from [44], where there is explanation on the abbreviated name choices.

The fact that those switches exhibit identical deterministic traces from specific initial conditions has a mathematical explanation. It was recently shown that a large class of complex systems with multiple positive feedback loops can emulate (the traces of) AM, where emulation in this context means that AM can summarize the key features of the larger systems (Fig 6) [44]. Structurally, emulation is based on homomorphism, which means that multiple reactions of a complex network can be collapsed into a lower number of reactions in a simpler system. For example, all reactions between species in the MI network can be related to the elements in the simpler network of AM (e.g., *z*_*2*_ and *y*_*0*_ in bottom wiring diagram [MI] collapse to *x*_*0*_ in the upper diagram [AM]; Fig 6). Furthermore, this structural homomorphism must satisfy a stoichiomorphism property that maintains the stoichiometric relationship between the elements. Despite the strong requirement on matching trajectories, there is a wide collection of systems with multiple feedback loops that can be mapped to AM (Fig 7). The most complex network shown is NCC, which is based on an extended version of the cell cycle switch [67]. In the presented case, all rates are taken equal to unity; more general situations are discussed next. Still, this means that, in this case, the highly complex NCC network can function equally well as AM to drive a biological switch.

In our examples, such as in Figs 3–5, we have taken all rates equal to unity (1.0), and we have typically considered simple initial conditions. These are simplifying assumptions that allow us to illustrate some trajectories that are representative of network behaviour and, in particular, illustrate at least the potential for a network to emulate another one. More generally, the trajectories of a network obviously depend on rates and initial conditions, sometimes critically so, and one should be rightfully sceptical of any particular choice of parameters. Questions about how much emulations are perturbed under perturbations of parameters can be very difficult to answer in general. Still, there are several factors that mitigate our arbitrary choices:

- All the reactions in our networks are bimolecular (because of our particular triplet motif, Fig 2). Hence, under any uniform scaling of the initial conditions, the kinetics remain the same up to scaling of the time axis (this would not hold if we had, for example, a mixture of bimolecular and monomolecular reactions). Although this property is specific to our triplets, it can hold for other uniform interpretations of influences.
- The definition of emulation (see Fig 6) requires that the trajectories of the two networks are aligned for all possible initial conditions of the target network (and for matching initial conditions of the source network). Yet, this property can be checked finitarily over the networks themselves [44]. Once we have found an emulation, we can choose to vary the initial conditions of the target network arbitrarily.
- If there is an emulation between two networks, that emulation does depend critically on the rates assigned to reactions in the two networks. For example, perturbing the rates in the source network will typically prevent an exact emulation of the target network, because the target network does not have as many degrees of freedom. Still, given an emulation, it is always possible to arbitrarily change the rates of the target network and find another emulation between those two networks by systematically changing the rates of the source network (Change of Rates Theorem in [44]). Hence, a unit-rate emulation entails emulations for many other rate assignments.
- The definition of emulation allows for networks with arbitrary rates, not just unit or uniform rates. It also allows for rate aggregation across the morphism: rates do not have to match exactly in the two networks but only achieve a certain kind of balance (which is the same as saying that only the resulting differential equations have to match).
- Influence networks in which each species has a single activation and a single inhibition (which covers all our examples) enjoy the following property: if there are no emulations between two unit-rate networks (and this can be checked finitarily), then there can be no emulations between those networks under any rate assignment [68]. This allows us to easily exclude the existence of emulations based on a check with unit rates.
- It is not always necessary to specify all rate parameters in the networks. We can leave some parameters undetermined and have a theorem prover search for them, looking for emulations. These computations can be carried out within decidable theories of arithmetic, where we always get a correct yes/no answer (given sufficient time) about existence of suitable parameters [69].
- In realistic examples, given realistic parameter sets, emulations will never hold exactly. Notions of emulations that are approximate up to some epsilon are being investigated and are a topic for future work. Meanwhile, it still seems useful to hold an exact emulation as a paradigm for a more realistic not-quite-exact emulation that may arise in a specific biological context, as we are demonstrating here.

The observation that—with appropriate parameter settings—complex networks can be collapsed to smaller ones (Fig 7) raises several questions. More complex networks have more possible behaviours, arising from their larger set of possible initial conditions. Still, if a complex network works as a switch, many of those possible behaviours may not be relevant. However, if that network can be collapsed into a simpler one without losing any of its switch-like properties, what are the benefits of evolving more complex networks to control biological switches?

Evolution is a tinkerer [70], genes are duplicated and specialized [71], and such processes could indeed drive evolution from AM to NCC, but it is not clear why the complexity of larger networks could be beneficial. Redundancy could add to the robustness of systems [52,72], but the dynamics of the complexly wired NCC could be also greatly perturbed by the removal of any components, because NCC does not contain parallel pathways; rather, it has longer feedback loops. Cells try to protect the networks that carry out important functions, but such networks should be flexible enough to include and interpret new stimuli. The growth in complexity then might arise neutrally and result in increased fitness and functional advantage by enabling the cell to respond to multiple different stimuli [73,74]. Complexity could also help to reduce noise in the system [75], although constraints on the extent to which this is possible have been demonstrated [76].

Recent comparison of several networks of Fig 7 revealed that intrinsic noise from molecular fluctuations is, indeed, reduced by more complex networks; even extrinsic noise from parameter perturbations is also greatly reduced in NCC compared to AM. The exact structure of the networks influences the amount of noise reduction [77], suggesting that evolution can find better solutions by increasing complexity.

As a final point, we note that, in AM and GW networks, the transient probability distributions of molecule copy numbers from the same initial conditions are different [26,77], while we observe that GW can emulate the deterministic dynamics of AM exactly (Fig 2). This also shows that, although emulation of the (deterministic) average behaviour could be perfect, networks with various levels of complexity might respond differently to the inherent molecular fluctuations of biological systems. This further highlights that the use of computer science approaches to investigate biological switches could eventually help us to understand how and why complex biological switches have evolved.

## Conclusion

We have discussed how an efficient population protocol (AM) exhibits similar dynamical features to a well-characterized biological switch (CC). This similarity allows us to conclude that evolution might have found an apparently complex but implicitly simple way of implementing efficient switches. These implicit patterns could have been used as building blocks to achieve efficient switching in a broad variety of wirings at various levels of complexity, demonstrating that, although system dynamics are important, the exact network structure and sequence of regulators can greatly differ.

Biological switches are known to be controlled by nonlinear positive feedback loops [78,79], and it is also known that multiple feedback loops make the switches even more robust [52,54]. Because these switches are highly conserved and reoccurring in various regulatory systems, we might think that they function optimally. Through comparison with an algorithm, we can find out if nature has picked an optimal or suboptimal solution to a problem. If the algorithm is computationally optimal (so that no other algorithm is faster up to a multiplicative constant), then we know that nature could not have done much better, and this may help explain why a specific network structure was selected. And we indeed know this is the case for AM. If the algorithm is not optimal, then either (A) our understanding is incomplete, (B) the problem does not require an optimal solution, or (C) the suboptimal algorithm is in practice faster than the optimal one in the operating regime (which is often the case for highly sophisticated optimal algorithms). Each of these possibilities provides insights. It is also interesting to consider how much evolution might select for speed in biological switches, but it is reasonable to assume that a slowly responding, inefficient system is counter selected.

Efficiency in biological contexts can be meant as speed to reach a stable state both in the sense of algorithmic efficiency and in a different sense as the use of limited resources in the establishment of a network. Sometimes the two are related: energy-limited minimal organisms might have used AM-like switches based on a single species (e.g., autocatalytic bifunctional enzymes), or MI/SI-like switches based on two antagonistic species (Fig 5B and 5C), to drive their responses to environmental changes. With the reduction in energy efficiency requirements, biological complexity could have neutrally increased, and, as evolution and engineers both select for dynamical effectiveness (reliability, robustness, and speed), the number of positive feedback loops could have increased in the system (three in AM versus eight in NCC on Fig 7). This increment could have facilitated the noise reduction efficiency of the switches and allowed the emergence of multiple regulatory pathways affecting the same switch in different ways.

Further investigation into the parallels between population protocols and biological systems might help us to better understand how and why complex signalling networks have evolved. Artificial biological switches were implemented in various cell types [80,81], and even the AM algorithm has been assembled in synthetic DNA computing systems [82]. Synthetic biology might provide the right tools [83,84] to investigate the efficiency of biological and computational switches in a controlled experimental setting. There is evidence of switching behaviour in various biological systems (some listed in Figs 3 and 5). So far, experimental testing has focussed more on identifying bistability by changing input parameters and scanning through a hysteresis loop [85,86] or moving out from steady state by intervention on key variables [87]. Following our simulations, it would be interesting to see if any of the discussed systems can be isolated and investigate if we see a quick transition to one or the other steady state and how this decision depends on initial settings.

We have seen that fundamental ideas can cross disciplines, and the findings of one discipline can be readily used to solve problems in another. As we have seen cross fertilizations in far, distinct fields, like how banking systems can be driven by rules learned from ecology [88], we believe that computer science and biology still have many insights to share. Both disciplines investigate paradigms such as robustness, efficiency, and reliability. Influence can go both ways [17,18]: biological findings can be used to improve the design of algorithms and theories, and computational concepts can help us use the increasing amount of experimental data to improve our understanding of complex biological systems.

## Supporting Information

S1 Text Simulation methods and codes. (PDF)
