# Supplementary material for: Efficient Switches in Biology and Computer Science
Source: PLoS Comput Biol. 2017 Jan 5;13(1):e1005100. doi: 10.1371/journal.pcbi.1005100 (PMC5215766; doi:10.1371/journal.pcbi.1005100)
Supplement: S1 Text — (PDF) [file pcbi.1005100.s001.pdf]

# S1 Text. Simulation methods and codes

# Efficient switches in biology and computer science

Luca Cardelli[1,2] Rosa D. Hernansaiz-Ballesteros[3] Neil Dalchau[1] & Attila Csikász-Nagy[3,4]

[1] Microsoft Research, 21 Station Road, Cambridge CB1 2FB, UK

[2] Department of Computer Science, University of Oxford, Wolfson Building, Parks Road, Oxford OX1 3QD, UK

[3] Randall Division of Cell and Molecular Biophysics, New Hunt's House, King's College London, London, SE1 1UL

[4] Pázmány Péter Catholic University, Faculty of Information Technology and Bionics, H-1083 Budapest, Hungary

### I. METHODOLOGY

The data to generate all time-course diagrams have been obtained by using Visual GEC software (http://lepton.research.microsoft.com/webgec/). The ODEs have been solved using a Runge-Kutta Method (RK547M), included in the software.

Graphics have been created by using ggplot2 package, from R.

All wiring diagrams of figures 3-7 and below are condensed versions. Each node (molecule) of each network represents the three states of the molecule: inactive form, non-decided form and active form. Thus, each node is showing up by three traces in simulation plots.

### II. LBS CODE FOR THE MODELS

| | | | |
|---|---|---|---|
| **AM** | Figure 3 | 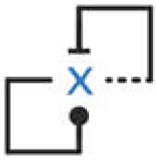 | directive sample 5 100<br>directive simulation deterministic<br><br>rate k = 1;<br><br>init x 1.001 \|<br>init xb 1 \|<br>init xi 1 \|<br><br>x + xi ->{k} xi + xb \|<br>xi + x ->{k} x + xb \|<br>xb + x ->{k} x + x \|<br>xb + xi ->{k} xi + xi |

| | | | |
|---|---|---|---|
| | Figure 5A | | directive sample 5 100<br>directive simulation deterministic<br><br>rate k = 1;<br><br>init x 2.001 \|<br>init xb 0 \|<br>init xi 2 \|<br><br>M + A  ->{k} M + U \|<br>A + x ->{k} M + U \|<br>U + x ->{k} M + M \|<br>U + A ->{k} A + A |
| **MI** | Figure 5B | 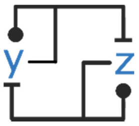 | directive sample 10.0 100<br>directive simulation deterministic<br><br>rate k = 1;<br><br>init z0 2.001 \|<br>init z1 0 \|<br>init z2 2 \|<br>init y0 2 \|<br>init y1 0 \|<br>init y2 2.001 \|<br><br>z2 + z0 ->{k} z0 + z1 \|<br>z1 + z0 ->{k} z0 + z0 \|<br>y0 + z0 ->{k} z0 + y1 \|<br>y1 + z0 ->{k} z0 + y2 \|<br><br>y2 + y0 ->{k} y0 + y1 \|<br>y1 + y0 ->{k} y0 + y0 \|<br>z0 + y0 ->{k} y0 + z1 \|<br>z1 + y0 ->{k} y0 + z2 |
| **SI** | Figure 5C | 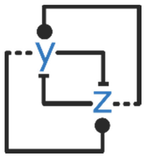 | directive sample 10.0 100<br>directive simulation deterministic<br><br>rate k = 1;<br><br>init z0 2.001 \|<br>init z1 0 \|<br>init z2 2 \|<br>init y0 2 \|<br>init y1 0 \|<br>init y2 2.001 \| |

| | | | |
|---|---|---|---|
| | | | z0 + y0 ->{k} y0 + z1 \|<br>z1 + y0 ->{k} y0 + z2 \|<br>z2 + y2 ->{k} y2 + z1 \|<br>z1 + y2 ->{k} y2 + z0 \|<br><br>y0 + z0 ->{k} z0 + y1 \|<br>y1 + z0 ->{k} z0 + y2 \|<br>y2 + z2 ->{k} z2 + y1 \|<br>y1 + z2 ->{k} z2 + y0 |
| **CC** | Figure 3 | 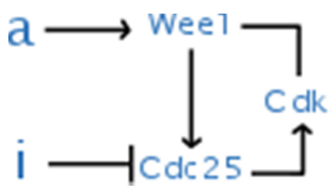 | directive sample 40 100<br>directive simulation deterministic<br><br>rate k = 1;<br><br>init CDK 1.001 \|<br>init CDC25 1.001 \|<br>init WEE1 1 \|<br>init CDKb 1 \|<br>init CDC25b 1 \|<br>init WEE1b 1 \|<br>init CDKi 1 \|<br>init CDC25i 1 \|<br>init WEE1i 1.001 \|<br>init a 1 \|<br>init i 1 \|<br><br>CDC25i + CDK ->{k} CDK + CDC25b \|<br>CDC25b + CDK ->{k} CDK + CDC25 \|<br>WEE1 + CDK ->{k} CDK + WEE1b \|<br>WEE1b + CDK ->{k} CDK + WEE1i \|<br><br>CDKi + CDC25 ->{k} CDC25 + CDKb \|<br>CDKb + CDC25 ->{k} CDC25 + CDK \|<br><br>CDK + WEE1 ->{k} WEE1 + CDKb \|<br>CDKb + WEE1 ->{k} WEE1 + CDKi \|<br><br>CDC25 + i ->{k} i + CDC25b \|<br>CDC25b + i ->{k} i + CDC25i \|<br>WEE1i + a ->{k} a + WEE1b \|<br>WEE1b + a ->{k} a + WEE1 |

<table>
<tr>
<td>GW</td>
<td>Figure 3</td>
<td>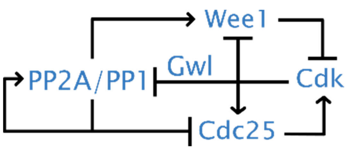
</td>
<td>directive sample 20 100<br>directive simulation deterministic<br><br>rate k = 1;<br><br>init CDK 1.001 |<br>init cdc25 1.001 |<br>init wee1 1 |<br>init PP1PP2A 1 |<br>init CDKb 1 |<br>init cdc25b 1 |<br>init wee1b 1 |<br>init PP1PP2Ab 1 |<br>init wee1i 1.001 |<br>init cdc25i 1 |<br>init CDKi 1 |<br>init PP1PP2Ai 1.001 |<br><br>cdc25i + CDK ->{k} cdc25b + CDK|<br>cdc25b + CDK ->{k} cdc25 + CDK|<br>PP1PP2A + CDK ->{k} PP1PP2Ab + CDK|<br>PP1PP2Ab + CDK ->{k} PP1PP2Ai + CDK|<br>wee1 + CDK ->{k} wee1b + CDK|<br>wee1b + CDK ->{k} wee1i + CDK|<br>CDK + wee1 ->{k} CDKb + wee1|<br>CDKb + wee1 ->{k} CDKi + wee1|<br>CDKi + cdc25 ->{k} CDKb + cdc25|<br>CDKb + cdc25 ->{k} CDK + cdc25|<br><br>PP1PP2Ai + PP1PP2A ->{k} PP1PP2Ab + PP1PP2A|<br>PP1PP2Ab + PP1PP2A ->{k} PP1PP2A + PP1PP2A|<br>wee1i + PP1PP2A ->{k} wee1b + PP1PP2A|<br>wee1b + PP1PP2A ->{k} wee1 + PP1PP2A|<br>cdc25 + PP1PP2A ->{k} cdc25b + PP1PP2A|<br>cdc25b + PP1PP2A ->{k} cdc25i + PP1PP2A</td>
</tr>
</table>

| | | | |
|---|---|---|---|
| **Extended GW** | Figure 4AC. No oscillations | 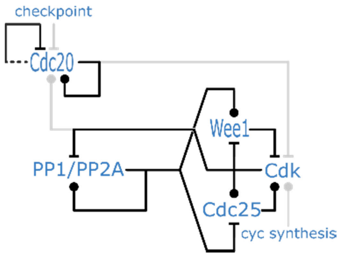 | directive sample 20 1000<br>directive simulation deterministic<br><br>rate k = 1;<br>rate k2 = 1;<br>rate re = 1;<br>rate k0 = 1;<br><br>init ch 3 \|<br>init cs 3 \|<br><br>init cdc20 3 \|<br>init cdc20i 4 \|<br>init cdc20b 2 \|<br><br>init cdk1 3 \|<br>init cdk1b 3 \|<br>init cdk1i 3 \|<br><br>init cdc25 3 \|<br>init cdc25i 3 \|<br>init cdc25b 3 \|<br><br>init wee1 3 \|<br>init wee1i 3 \|<br>init wee1b 3 \|<br><br>init PP1PP2A 3 \| |

<table>
<tr>
<td></td>
<td></td>
<td></td>
<td>init PP1PP2Ab 3 |<br>init PP1PP2Ai 3 |<br><br>wee1i + PP1PP2A ->{k} wee1b + PP1PP2A|<br>wee1b + PP1PP2A ->{k} wee1 + PP1PP2A|<br>wee1 + cdk1 ->{k0} wee1b + cdk1|<br>wee1b + cdk1 ->{k0} wee1i + cdk1|<br><br>cdc25i + cdk1 ->{k0} cdc25b + cdk1|<br>cdc25b + cdk1 ->{k0} cdc25 + cdk1|<br>cdc25 + PP1PP2A ->{k} cdc25b + PP1PP2A|<br>cdc25b + PP1PP2A ->{k} cdc25i + PP1PP2A|<br><br>PP1PP2A + cdk1 ->{k} PP1PP2Ab + cdk1|<br>PP1PP2Ab + cdk1 ->{k} PP1PP2Ai + cdk1|<br>PP1PP2Ai + PP1PP2A ->{k} PP1PP2Ab + PP1PP2A|<br>PP1PP2Ab + PP1PP2A ->{k} PP1PP2A + PP1PP2A|<br><br>cdk1i + cdc25 ->{k0} cdk1b + cdc25|<br>cdk1b + cdc25 ->{k0} cdk1 + cdc25|<br>cdk1i + cs ->{k0} cdk1b + cs |<br>cdk1b + cs ->{k0} cdk1 + cs |<br>cdk1 + wee1 ->{k0} cdk1b + wee1|<br>cdk1b + wee1 ->{k0} cdk1i + wee1|<br>cdk1 + cdc20 ->{re} cdk1b + cdc20|<br>cdk1b + cdc20 ->{re} cdk1i + cdc20|<br><br>cdc20i + cdk1 ->{re} cdc20b + cdk1|<br>cdc20b + cdk1 ->{re} cdc20 + cdk1|<br>cdc20 + ch ->{re} cdc20b + ch|<br>cdc20b + ch ->{re} cdc20i + ch|<br>cdc20i + cdc20 ->{k0} cdc20b + cdc20|<br>cdc20b + cdc20 ->{k0} cdc20 + cdc20|<br>cdc20 + cdc20i ->{re} cdc20b + cdc20i |<br>cdc20b + cdc20i ->{re} cdc20i + cdc20i</td>
</tr>
<tr>
<td>Extended GW</td>
<td>Figure 4BD. Oscillations</td>
<td>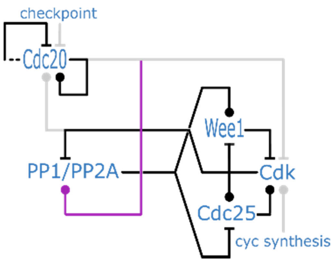
</td>
<td>directive sample 20 1000<br>directive simulation deterministic<br><br>rate k = 1;<br>rate k2 = 1;<br>rate re = 1;<br>rate k0 = 1;<br><br>init ch 3 |<br>init cs 3 |<br><br>init cdc20 3 |<br>init cdc20i 4 |<br>init cdc20b 2 |<br><br>init cdk1 3 |</td>
</tr>
</table>

| | | | |
|---|---|---|---|
| | | | init cdk1b 3 \|<br>init cdk1i 3 \|<br><br>init cdc25 3 \|<br>init cdc25i 3 \|<br>init cdc25b 3 \|<br><br>init wee1 3 \|<br>init wee1i 3 \|<br>init wee1b 3 \|<br><br>init PP1PP2A 3 \|<br>init PP1PP2Ab 3 \|<br>init PP1PP2Ai 3 \|<br><br>wee1i + PP1PP2A ->{k} wee1b + PP1PP2A\|<br>wee1b + PP1PP2A ->{k} wee1 + PP1PP2A\|<br>wee1 + cdk1 ->{k0} wee1b + cdk1\|<br>wee1b + cdk1 ->{k0} wee1i + cdk1\|<br><br>cdc25i + cdk1 ->{k0} cdc25b + cdk1\|<br>cdc25b + cdk1 ->{k0} cdc25 + cdk1\|<br>cdc25 + PP1PP2A ->{k} cdc25b + PP1PP2A\|<br>cdc25b + PP1PP2A ->{k} cdc25i + PP1PP2A\|<br><br>PP1PP2A + cdk1 ->{k} PP1PP2Ab + cdk1\|<br>PP1PP2Ab + cdk1 ->{k} PP1PP2Ai + cdk1\|<br>PP1PP2Ai + cdc20 ->{k0} PP1PP2Ab + cdc20\|<br>PP1PP2Ab + cdc20 ->{k0} PP1PP2A + cdc20\|<br><br>cdk1i + cdc25 ->{k0} cdk1b + cdc25\|<br>cdk1b + cdc25 ->{k0} cdk1 + cdc25\|<br>cdk1i + cs ->{k0} cdk1b + cs \|<br>cdk1b + cs ->{k0} cdk1 + cs \|<br>cdk1 + wee1 ->{k0} cdk1b + wee1\|<br>cdk1b + wee1 ->{k0} cdk1i + wee1\|<br>cdk1 + cdc20 ->{re} cdk1b + cdc20\|<br>cdk1b + cdc20 ->{re} cdk1i + cdc20\|<br><br>cdc20i + cdk1 ->{re} cdc20b + cdk1\|<br>cdc20b + cdk1 ->{re} cdc20 + cdk1\|<br>cdc20 + ch ->{re} cdc20b + ch\|<br>cdc20b + ch ->{re} cdc20i + ch\|<br>cdc20i + cdc20 ->{k0} cdc20b + cdc20\|<br>cdc20b + cdc20 ->{k0} cdc20 + cdc20\|<br>cdc20 + cdc20i ->{re} cdc20b + cdc20i \|<br>cdc20b + cdc20i ->{re} cdc20i + cdc20i |
